# Direct medical costs attributable to type 2 diabetes mellitus: a population-based study in Catalonia, Spain

**DOI:** 10.1007/s10198-015-0742-5

**Published:** 2015-11-05

**Authors:** Manel Mata-Cases, Marc Casajuana, Josep Franch-Nadal, Aina Casellas, Conxa Castell, Irene Vinagre, Dídac Mauricio, Bonaventura Bolíbar

**Affiliations:** 1 Primary Health Care Center La Mina, Institut Català de la Salut, Sant Adrià de Besòs, Spain; 2 Research Support Unit Barcelona Ciutat, Primary Healthcare Research Institute Jordi Gol (IDIAP Jordi Gol), Barcelona, Spain; 3 CIBER of Diabetes and Associated Metabolic Diseases (CIBERDEM), Barcelona, Spain; 4 Primary Healthcare Research Institute Jordi Gol (IDIAP Jordi Gol), Barcelona, Spain; 5 Universitat Autònoma de Barcelona, Barcelona, Spain; 6 Primary Health Care Center Raval, Institut Catala de la Salut, Barcelona, Spain; 7 Public Health Management Agency, Department of Health, Generalitat de Catalunya, Barcelona, Spain; 8 Department of Endocrinology and Nutrition, Diabetes Unit, Hospital Clinic, University of Barcelona, Barcelona, Spain; 9 Department of Endocrinology and Nutrition, Health Sciences Research Institute, University Hospital Germans Trias i Pujol, Carretera Canyet S/N, 08916 Badalona, Barcelona Spain

**Keywords:** Type 2 diabetes, Costs, Primary care, Retrospective, Population database, Catalonia, Spain, I1, H75

## Abstract

We estimated healthcare costs associated with patients with type 2 diabetes compared with non-diabetic subjects in a population-based primary care database through a retrospective analysis of economic impact during 2011, including 126,811 patients with type 2 diabetes in Catalonia, Spain. Total annual costs included primary care visits, hospitalizations, referrals, diagnostic tests, self-monitoring test strips, medication, and dialysis. For each patient, one control matched for age, gender and managing physician was randomly selected from a population database. The annual average cost per patient was €3110.1 and €1803.6 for diabetic and non-diabetic subjects, respectively (difference €1306.6; i.e., 72.4 % increased cost). The costs of hospitalizations were €1303.1 and €801.6 (62.0 % increase), and medication costs were €925.0 and €489.2 (89.1 % increase) in diabetic and non-diabetic subjects, respectively. In type 2 diabetic patients, hospitalizations and medications had the greatest impact on the overall cost (41.9 and 29.7 %, respectively), generating approximately 70 % of the difference between diabetic and non-diabetic subjects. Patients with poor glycaemic control (glycated haemoglobin >7 %; >53 mmol/mol) had average costs of €3296.5 versus €2848.5 for patients with good control. In the absence of macrovascular complications, average costs were €3008.1 for diabetic and €1612.4 for non-diabetic subjects, while its presence increased costs to €4814.6 and €3306.8, respectively. In conclusion, the estimated higher costs for type 2 diabetes patients compared with non-diabetic subjects are due mainly to hospitalizations and medications, and are higher among diabetic patients with poor glycaemic control and macrovascular complications.

**Electronic supplementary material:**

The online version of this article (doi:10.1007/s10198-015-0742-5) contains supplementary material, which is available to authorized users.

## Introduction

Type 2 diabetes mellitus (T2DM) is a global health problem due to its high worldwide prevalence, high cost of treatment, associated chronic complications, disability, and premature deaths [1]. Its incidence and prevalence in developed countries has increased in recent decades, and several studies confirm that this increase will continue [1–4]. This rising prevalence, together with a progressively aging population, will significantly increase the use of healthcare products and services in the near future [5]. Moreover, chronic complications of T2DM have a well-known negative impact on costs [3, 6, 7] and patients’ quality of life [1, 6, 8].

The high cost of managing T2DM and its associated complications represents a major economic burden for healthcare organizations at both the primary and specialized level [9]. A recent study conducted in the United States (US) showed that around 20 % of medical costs are generated by complications associated with the disease [5]. Among acute complications, severe hypoglycaemia has the highest cost for the health system [10]. However, late complications, especially end stage renal disease (ESRD) requiring dialysis and transplantation, have the greatest impact on morbidity and mortality [11, 12]. This was confirmed in a recent study in the US in which cardiovascular disease was associated with cost increases of between 70 % and 150 %, and ESRD with increases up to 500 % [12].

Previous research also indicates that medical costs per person are much higher in patients with diabetes than in the general population [13, 14]—a difference due mainly to the increased use of hospital resources caused by complications of the disease [1, 5, 12–18]. The results of country-specific estimates for Spain from the Cost of Diabetes in Europe–Type 2 (CODE-2) study showed an average annual healthcare cost per diabetic patient of €1305 in 1999 (equivalent to around €3363 in 2011), including direct costs [15], with medication, hospitalization and primary care visits accounting for most of this figure. There was a 1.6-fold increase in patients with microvascular complications, and a 2.3-fold increase in patients with macrovascular complications [15].

In Europe, the few studies that have compared healthcare costs between diabetic and non-diabetic populations have shown an increase in costs of around 60–80 % [13, 14, 19]. However, these studies have some methodological limitations that make comparisons between groups (diabetic vs. non-diabetic patients) difficult, and hinder the establishment of the costs attributable to type 2 diabetes with certainty: they included both type 1 and type 2 diabetic patients; they excluded treatment with drugs other than antidiabetic agents; and they were not matched for age, gender and managing physician.

The present study was conducted to estimate the resource consumption and additional costs attributable to T2DM for the Spanish National Health System in 2011 in comparison with a control group of non-diabetic subjects matched for age, gender and managing physician, selected randomly from a population database.

## Methods

### Research design and subjects

The present Diabetes Mellitus Cost Study (eCostesDM) is a retrospective study designed to estimate and compare the costs of patients with T2DM with those of non-diabetic patients treated at primary care centers in the Catalan Health Institute (ICS) system in 2011.

### Patient selection

All patients aged between 31 and 90 with a diagnosis of T2DM [International Classification of Diseases (ICD) codes E11, E14] seen in primary care in 2011 were included, and all variables recorded during 2011 were entered in the study following the same criteria as previous studies [20–22]. Subsequently, an individual matching was applied in which, for each diabetic patient, one patient not diagnosed with diabetes was included, and matched on gender, age (maximum difference of 2 years), and managing physician. For this, we used a simple random sampling without replacement: all non-diabetic patients matching the individual diabetic patient were selected, and a random number was assigned to each of them. After this, the subject with the lowest random number was chosen, and removed from the control population so that it could not be selected again. Subjects in either group who were not alive at the end of the study were not included in the analyses.

### Information and variables of interest

Data were extracted from the Information System for the Development of Research in Primary Care (SIDIAP) database, which is intended for use in research and includes the e-CAP medical records of all patients in the Catalan Health Institute [23, 24].

The Catalan Health Institute (Institut Català de la Salut—ICS) is the main provider of primary healthcare services in Catalonia. It manages 470 primary care teams caring for more than 5.5 million citizens, approximately 83 % of the Catalan population. All ICS primary care professionals (about 15,000) use the same computerized medical record program (e-CAP), created and managed by the institution itself. The ICS also directly manages 8 hospitals while the remaining 58 hospitals in Catalonia are members of the Network of Hospitals of Public Utilization (XHUP) (http://www.gencat.cat/ics/english.htm, last accessed 15 July 2015).

To estimate the consumption of healthcare resources we used the SIDIAP^Q^ subpopulation, which is composed of those patients with the most complete medical histories [23, 24], and includes data from 1,878,816 of the 5.8 million patients registered in the parent SIDIAP database. This information was supplemented with data registered in the *ConjuntoMínimoBásico de Datos de AltasHospitalarias* (CMBD_HA; Set of Minimum Basic Data Set of Hospital Admissions) [25], which records all hospital admissions in Catalonia. The CMBD follows the recommendations of the European Minimum Basic Data Committee, and contains information on clinical records and discharge reports from hospitals. However, it does not have information about medical emergencies, including hypoglycemia or visits to the hospital outpatient’s services.

The analysis included healthcare direct costs of diabetic patients for the National Health Service. Data on private healthcare costs for the patients themselves or for their relatives were not available.

The following direct costs were analyzed in the study: primary care visits (differentiating between doctor’s or nurse’s visits, and between place of visit, i.e., in the office or at home), hospitalizations, referrals to specialist care, diagnostic tests, medication, dialysis treatment, and use of self-monitoring test strips. The antidiabetic treatment variable included three categories: non-pharmacologic treatment, treatment with non-insulin antidiabetic drugs only, and treatment with insulin (with or without other antidiabetic agents). Finally, we did not impute missing data or modeled censored data. Patients with no registered costs (not using healthcare resources during the study period) were not excluded.

### Cost calculation

To calculate the costs we used the prices established in the Official Bulletin of the Catalan Government (DOGC) for 2012 [26]. All costs are expressed in Euros at 2011 prices. For this, the prices set by the Department of Health were adjusted to 2011 values by subtracting 0.9 % to account for inflation [27].

The costs of hospital admissions were obtained using the Diagnosis-Related Groups (DRG) patient classification system [28] published in the DOGC [26], and weighted by level of hospital complexity (high, medium, and low). For medications, the retail price was used based on the pharmacy billing information. For self-monitoring test strips, we used the mean cost per patient in each primary care team. For the cost of dialysis, the mean frequency was estimated to be three sessions a week. As information on the type of dialysis used for each patient was not available, the cost of each session was weighted according to local use (20 % peritoneal dialysis, and 80 % haemodialysis) [29], using the prices established by the DOGC. All the variables and information on their sources and costs are shown in the Online Resource (Table S1).

The final costs of the two study populations were calculated as follows: the resources consumed by each individual under each of the resource variables were quantified, and then multiplied by the cost of each resource. This procedure yielded the total resource cost per individual as well as the overall cost for each study population (diabetic subjects vs. subjects without diabetes).

### Sensitivity analysis

We performed a deterministic sensitivity analysis by modifying the value of cost variables corresponding to hospitalizations and primary care visits. For hospitalizations, two different calculation methods were used: the DRG method [28], and the combination of relative intensity of structure (RIS) and relative intensity of resources (RIR), which weights the complexity of the structure and the resources to estimate the cost of a hospital discharge [30]. For the primary care visit cost variable, two methods were used: one based on the public system costs published in the DOGC, and the other on the private costs provided by the Oblikue database [31] (Online Resource, Table S1). To assess the difference between groups based on prices used we compared the ratios between T2DM patients and subjects without diabetes.

### Statistical analysis

We compared the mean total cost of resource consumption, and the mean cost of each individual resource consumed according to the presence or absence of T2DM. The Generalized Linear Models (GLM) methodology was used to study the effect of T2DM on cost, as well as the factors associated with increased costs in T2DM patients. Given the asymmetry of the cost variable, we used the Gamma family and the logarithm as the link function [32].

In the first model, in which we compared diabetic versus non-diabetic subjects, we used a GLM model with four steps: first the raw effect of T2DM; second the adjusted effect for gender and age; third the adjusted effect for gender, age, cardiovascular disease, and heart failure; and fourth adding end-stage renal failure (stage 5) or dialysis to the third model. For the second analysis, considering only diabetic patients, a GLM model was estimated including age, gender, duration of T2DM, antidiabetic treatment, cardiovascular disease, heart failure, kidney disease, microvascular complications, obesity, and glycated hemoglobin HbA1c values. To deal with the high occurrence of zeros in the observed data (non diabetic group), we used a hurdle model to count positive outcomes (>0), using truncated negative binomial regression. Analyses were performed using SPSS (released 2009. PASW Statistics for Windows, Version 18.0. SPSS Inc., Chicago IL) and StataCorp. 2013. (Stata Statistical Software: Release 13. StataCorp LP, College Station, TX). For the hurdle model, we used the R-3.2.2 Package ‘pscl’ [33].

## Results

The mean ages of the non-diabetic and T2DM populations were 67.5 years [standard deviation (SD) = 11.6], and 67.6 years (SD = 11.7), respectively. In both groups, slightly over half (53.5 %) were men. The median duration of T2DM was 6.2 years (interquartile range 3.2–9.6), and a mean of 7.2 years (SD = 5.8). Patients with T2DM were on average more obese, and had more hypertension and diabetes-related conditions than the control group (Table 1). Consumption of all resources was increased in the T2DM group (Table 1).

**Table 1:** Demographic and clinical characteristics, and consumption of healthcare resources in patients with type 2 diabetes mellitus (T2DM) or without diabetes

	Patients with T2DM (*n* = 126,811)	Patients without diabetes (*n* = 126,811)
Age, mean (SD), years	67.6 (11.7)	67.5 (11.6)
Men (%)	53.5	53.5
Diabetes duration, median (interquartile range), years	6.2 (3.2–9.6)	–
HbA1c, mean (SD), % (*n* = 100,391)	7.1 (1.4)	–
HbA1c, mean (SD), mmol/mol, (*n* = 100,391)	54.1 (12.9)	–
BMI (kg/m^2^)	30.4 (5.3)	28.6 (5.3)
Systolic blood pressure, mean (SD), mmHg	137 (13.7)	134 (13.7)
Diastolic blood pressure, mean (SD), mmHg	76 (8.8)	76 (8.8)
Hypertension (%)	68.7	45.9
LDL-cholesterol, mean (SD), mg/dL	111.5 (32.7)	130.1 (32.2)
Smoking habit (%)	14.8	16.6
Antidiabetic treatment		
No pharmacological treatment (%)	26.9	–
Non-insulin antidiabetic drugs (%)	56.2	–
Insulin (alone or with other antidiabetic drugs) (%)	16.8	–
Complications		
Diabetic retinopathy (%)	7.2	–
Coronary heart disease (%)	12.7	6.4
Stroke (%)	7.5	4.7
Peripheral artery disease (%)	4.6	1.8
Diabetic neuropathy (%)	21.0	–
Heart failure (%)	5.7	2.8
Chronic renal failure (eGFR <60 mL/min/1.73 m^2^)^a^ (%)	18.8	16.6
Microalbuminuria (30–300 mg/mL)^b^ (%)	13.6	7.6
Macroalbuminuria (>300 mg/mL)^b^ (%)	2.1	0.8
Use of healthcare resources		
Primary care visits, mean (SD)	16.3 (15.3)	10.1 (11.9)
Hospitalizations^c^, mean (SD), days	8.2 (13.3)	6.7 (11.5)
Referrals, mean (SD)	1.0 (1.4)	0.7 (1.1)
Diagnostic tests, mean (SD)	0.3 (0.5)	0.2 (0.6)
Laboratory parameters, mean (SD)	19.1 (18.5)	10.88 (14.4)

*BMI* Body mass index, *eGFR* estimated glomerular filtration rate using the MDRD (Modified Diet in Renal Disease) formula

^a^Patients with eGFR available: with type 2 diabetes 90,195; without diabetes 65,174

^b^Patients with albuminuria available: with type 2 diabetes 57,886; without diabetes 18,214

^c^Hospitalized patients: with type 2 diabetes 32,154; without diabetes 19,204

The estimated annual cost per person in the T2DM group was €3110.1 [95 % confidence interval (CI) 3065.8–3154.3, median €1548.2; 25 percentile: €825.5; 75 percentile: €2883.4] and €1803.4 (95 % CI 1759.6–1847.3, median €730; 25 percentile: €261; 75 percentile: €1601) in the non-diabetic group. The annual cost was 72.4 % higher in the T2DM group (Table 2). The variables with the greatest impact on the overall cost in the T2DM group were hospitalizations (41.9 %), medications (29.7 %), and primary care visits (18.5 %). Hospitalizations and medications generated approximately 70 % of the difference in total annual cost between the two groups. The two other variables presenting large differences between groups were specialists’ referrals and diagnostic tests, although they represented a small proportion of expenditure.

**Table 2:** Mean annual direct medical cost (95 % CI) in patients with T2DM and without diabetes for each of the major cost categories

	Patients with T2DM (*n* = 126,811)	Patients without diabetes (*n* = 126,811)	Difference between means	Increase in cost (%)
Total annual direct cost (€)	3110.1 (3065.8–3154.3)	1803.6 (1759.6–1847.3)	1306.6 (1244.3–1368.9)	+72.4
Primary care visits (€)	577.0 (573.9–580.0)	369.1 (366.7–371.4)	207.9 (204.0–211.9)	+56.3
Hospitalizations (€)	1303.1 (1262.0–1344.0)	801.6 (758.9–844.2)	501.4 (442.3–560.6)	+62.9
Referrals (€)	114.8 (113.9–115.7)	77.5 (76.8–78.3)	37.3 (36.2–38.4)	+48.1
Diagnostic tests (€)	82.4 (81.8–83.1)	46.5 (45.9–46.9)	35.9 (35.2–36.8)	+77.2
Self-monitoring blood test strips (€)	50.1 (49.9–50.1)	0	50.1 (49.99–50.12)	–
Medication (€)	925.0 (919.2–930.8)	489.2 (485.0–493.5)	435.8 (428.6–443.0)	+89.1
Dialysis (€)	57.8 (51.0–64.6)	19.7 (15.7–23.7)	38.1 (30.2–46.0)	+193.4

According to gender (Online Resource, Table S2), total annual costs were higher in men, especially in the T2DM group: €3143.8 (95 % CI 3080.2–3207.4) in men vs. €3071.2 (95 % CI 3010.5–3132.0) in women. The variable with the greatest gender differences between the two groups were hospitalizations, with an increase in costs in the T2DM group of €246.9 (95 % CI 472.6–621.6) in men and €448.9 (95 % CI 354.9–542.9) in women.

There was a progressive increase in costs with increase in age for all age groups, although in non-diabetic patients the increase was more pronounced (Online Resource, Table S3, and Fig. S1). The differences in costs between patients with and without T2DM narrowed progressively with increasing age (Online Resource, Fig. S1). As for the degree of glycaemic control (Table 3), diabetic patients with poor control (HbA1c >7 %; >53 mmol/mol) had increased costs of €448.0/patient/year (95 % CI 365.7–530.4) compared to those with good control. The two main variables causing this increase were medications €232.9 (95 % CI 220.5–245.3) and hospitalizations €170.4 (95 % CI 92.5–248.2).

**Table 3:** Mean annual direct medical cost (95 % CI) in patients with T2DM according to the degree of glycaemic control

	HbA1c >7 % (>53 mmol/mol) (*n* = 42,632)	HbA1c ≤7 % (≤53 mmol/mol) (*n* = 57,759)	Difference between good/bad control^a^
Total annual direct cost (€)	3296.5 (3229.6–3363.5)	2848.5 (2797.9–2899.0)	448.0 (365.7–530.4)
Primary care visits (€)	329.7 (326.2–333.3)	302.8 (299.9–305.8)	26.9 (22.3–31.5)
Hospitalizations (€)	1301.6 (1239.0–1364.2)	1131.2 (1084.9–1177.5)	170.4 (92.5–248.2)
Referrals (€)	128.4 (126.8–130.1)	120.0 (118.7–121.3)	8.4 (6.4–10.5)
Diagnostic tests (€)	92.2 (91.3–93.1)	88.2 (87.4–88.9)	4.0 (2.9–4.7)
Self-monitoring blood test strips (€)	50.0 (49.9–50.1)	49.9 (49.8–50.0)	0.1 (−0.1 to +0.2)
Medication (€)	1064.1 (1054.3–1073.8)	831.2 (823.5–838.8)	232.9 (220.5–245.3)
Dialysis (€)	23.7 (16.2–31.1)	41.9 (33.3–50.5)	−18.2 (−6.8 to +29.6)

^a^Good control was defined as HbA1c ≤7 % (≤53 mmol/mol)

Table S4 in the Online Resource shows the effect of comorbidity and diabetes complications on the increase in cost per patient. Costs were significantly higher in the T2DM group for all conditions except micro- and macroalbuminuria for which the costs were higher (though not significantly) in the non-diabetes group. In the absence of cardiovascular disease, the cost was €3008.1 (95 % CI 2963.8–3052.3) in diabetic patients and €1612.4 (95 % CI 1566.0–1658.7) in non-diabetic subjects, while the presence of any cardiovascular disease increased costs to €4814.6 (95 % CI 4683.7–4947.4) and €3306.8 (95 % CI 3173.0–3440.6), respectively. The complication that produced the greatest increase was heart failure, reaching €6866.6 (95 % CI 6563.1–7170.1) per year in patients with T2DM compared to €4759.2 (95 % CI 4422.6–5095.8) in non-diabetic subjects; this difference of €2107.4 (95 % CI 1654.2–2560.6) was the largest difference in costs between the two groups. As regards antidiabetic treatment, 26.9 % of diabetic patients received no medication (only lifestyle intervention), 35.4 % oral monotherapy, 20.2 % oral combination therapy and 16.5 % insulin either alone or in combination with non-insulin antidiabetic medication. The cost of antidiabetic drugs was €222/patient/year (24 % of the cost of pharmaceutical prescriptions, and 7.1 % of the total cost).

Estimation of the crude effect of T2DM on healthcare costs through a multivariate hurdle model (Online Resource, Table S5) indicated that costs were 54 % higher in diabetic patients than in non-diabetic subjects. Adjusting for sociodemographic and clinical variables (age, gender, cardiovascular disease, heart failure, and end stage renal disease), the impact of diabetes on healthcare costs decreased slightly by 13 %, but remained significantly increased (41 %). This model took into account the difference in the number of patients with zero direct medical costs (2 diabetic patients vs. 14,131 non-diabetic subjects).

In the multivariate analysis (GLM model) including only diabetic patients (Table 4), the variables with the greatest impact on cost were renal failure, with costs more than 10 times higher for patients in stage 5 (eGFR <15 or dialysis), and heart failure, which increased costs by almost 70 %. Treatment with antidiabetic drugs also increased costs by 11 % in non-insulin-treated and 64 % in insulin-treated patients. Costs were 42 % higher in patients with cardiovascular disease than in those without. Finally, costs also increased with age in both T2DM and non-diabetic groups, but the effect of T2DM gradually decreased; the difference was the largest in patients aged under 46 years (quotient 3.5) and fell progressively to only 1.4 in those aged over 80 (Online Resource, Table S5 and Fig. 1).

**Table 4:** Multivariate analysis. Variables influencing the direct medical cost in patients with T2DM.* eGFR* Estimated glomerular filtration rate by modification of diet in renal disease (MDRD)

	Odds ratio (95 % CI)	*P* value
Age, years		
<45	1.00	
46–55	1.22 (1.12–1.33)	<0.001
56–65	1.21 (1.12–1.32)	<0.001
66–80	1.18 (1.09–1.27)	0.001
≥81	1.25 (1.15–1.36)	<0.001
Gender		
Male	1.00	
Female	0.92 (0.90–0.95)	<0.001
T2DM duration		
≤10 years	1.00	
>10 years	1.06 (1.02–1.10)	0.001
Treatment of T2DMs		
No pharmacological treatment	1.00	
Non-insulin antidiabetic agents	1.11 (1.07–1.15)	<0.001
Insulin^a^	1.64 (1.56–1.72)	<0.001
Cardiovascular disease^b^	1.42 (1.37–1.47)	<0.001
Heart failure	1.69 (1.59–1.80)	<0.001
Kidney disease		
Stages 1–3	1.00	
Stage 4 (eGFR <30 mL min^−1^ 1.73 m^−2^)	1.56 (1.36–1.79)	<0.001
Stage 5 (eGFR <15 mL min^−1^ 1.73 m^−2^ or dialysis)	10.35 (6.96–15.40)	<0.001
Microvascular complications^c^	1.18 (1.13–1.22)	<0.001
Obesity	1.10 (1.07–1.13)	<0.001
Glycaemic control		
HbA1c ≤7 % (≤53 mmol mol^−1^)	1.00	
HbA1c 7.01–8 % (53.1–63.9 mmol mol^−1^)	1.00 (0.97–1.04)	0.786
HbA1c 8.01–9 % (64–74.9 mmol mol^−1^)	1.05 (1.00–1.10)	0.052
HbA1c 9.01–10 % (75–85.8 mmol mol^−1^)	1.01 (0.94–1.07)	0.880
HbA1c >10 % (>85.8 mmol mol^−1^)	1.08 (1.01–1.16)	0.032

^a^Insulin alone or in combination with non-insulin antidiabetics

^b^Includes peripheral artery disease, stroke, and coronary heart disease

^c^Includes diabetic retinopathy, diabetic nephropathy (micro or macroalbuminuria), and diabetic neuropathy

In the sensitivity analysis, hospital expenditure was reduced by approximately 50 % in both groups when using the combination of RIS and RIR method compared to the DRG method. However, the ratios between T2DM patients and subjects without diabetes using either price were almost identical (1.63:1 vs. 1.60:1). In the sensitivity analysis of the primary care visit costs, the cost decreased in both groups when using the Oblikue prices [26, 31] compared to the costs published in the DOGC, but the ratio between groups remained practically unchanged (1.56:1 vs. 1.62:1).

## Discussion

This article reports the costs attributable to T2DM in the primary care population in Spain. In 2011, the mean annual cost in patients with T2DM was 72.4 % greater than in non-diabetic subjects. The cost per patient is similar to that reported in previous studies in Spain. For example, after conversion to 2011 Euros, the annual costs reported in the 1998 CODE-2 study were €3363 [15], €3736 in the Ballesta et al. study in 1999 [3], €1610 in the Spain Estimated cost Ciberdem-Cabimer in Diabetes (SECCAID) study in 2010 [34], and €2229 in the study by Sicras-Mainar et al. in 2012 [19]. The SECCAID figure is notably lower, probably due to the different methodology used (top-down), where the total expenditure for the health system was divided by the total estimated number of T2DM patients [34]. In our study and in those by Ballesta et al., CODE-2, and Sicras-Mainar et al. data were obtained directly from patient records [3, 15, 19].

A cost comparison with other countries is difficult because of differences between healthcare systems and methodologies used. In Table S6 (Online Resource), the results of studies that compared the costs in diabetic and non-diabetic patients are shown. Costs recorded in two studies conducted outside Spain, converted to 2011 €, are notably higher: €4519 in Italy in 2003 [14], and €8117 in the US in 2012 [5]. The Italian study [14] included items corresponding to emergency care systems, and the US study [5] included indirect costs related with mortality and lost productivity, beyond the fact that the healthcare system is mostly private. Finally, both studies included patients with type 1 diabetes, and did not differentiate costs between T2DM and type 1 diabetes. In our study, the impact of the disease on costs was higher in men, in the 66–80 years age group, and in patients with complications. This is in accordance with previous research showing that costs may be substantially increased by cardiovascular and microvascular complications [11, 35], especially ESRD requiring dialysis and transplantation [35].

As in previous studies [2, 3, 7, 13, 14], the distribution of costs by components was quite similar between diabetic and non-diabetic subjects: the highest proportion of the costs was distributed between hospital care, medication, and primary care.

Previous studies comparing the costs of diabetic versus non-diabetic populations have reported cost ratios considerably higher than ours [5, 13, 14, 16–18, 36] (Online Resource, Table S6), with the exception of the study by Wiréhn et al. [13], in which the difference is only slightly higher. In our study the cost of diabetic patients was 1.54 times higher than the cost of non-diabetics subjects, and this ratio dropped to 1.41 after adjustment for contributing variables, namely age, gender, presence of cardiovascular disease, heart failure, and dialysis or transplantation. This ratio is quite below the 1.8 reported in a Swedish cost analysis published in 2005 [13]. However, that study did not differentiate between patients with type 1 diabetes and type 2 diabetes; the ratio was only 1.36 in patients over 75 years, similar to the rate observed in our study (1.95 in the over-80 age group), while in children it was 7.69 [13]. In an Italian study (the Turin Study, 2004) [14], the cost of medication per person and year were 2.8 times higher in diabetic patients, and represented 18.5 % of the total care costs. Medication costs were 7.7 times higher in type 1 diabetes patients, and 2.5 times higher in T2DM patients compared with non-diabetic subjects [14]. In Germany, in two studies published by Köster et al. in 2001 and 2010 [16, 17], the cost ratio was the same in both groups, i.e. 1.9, quite similar to our results (Online Resource, Table S6). In contrast, results from studies performed in the US in 2007 and 2012 showed cost ratios of 3 and 2.9, respectively [5, 18].

A recently published study in the city of Badalona (Catalonia, Spain) compared the costs of 3760 patients with T2DM in a total of 26,845 patients managed at six primary care centers and followed up prospectively during 2011 and 2012 [19]. The total costs of patients with T2DM after 2 years was €4458 compared with €2784 in non-diabetic subjects, with a ratio (1.6) very similar to that observed in our study (1.77). Although the total annual cost observed in that study (€2229) was lower than in ours, possibly due to differences in the methodology used for the allocation of costs, the 60 % increase in the cost attributable to the disease is similar to our results [19].

Regarding the impact of antidiabetic therapy, we must stress that about a quarter of the patients in our study were not receiving medication, and 35.4 % were receiving monotherapy (usually metformin), and only 16.5 % were receiving insulin. Therefore, the cost of antidiabetic drugs was lower than in other studies and represented only 22 % of the pharmacy costs. By comparison, for example, in the Italian study [14], 26.2 % of patients were treated with insulin, and 20.9 % were receiving non-pharmacological treatment. In the Swedish study discussed above [13], the percentage of patients receiving insulin is not mentioned; however, the difference in costs for patients receiving insulin suggests that it was high; in fact in the country-specific Swedish section of the CODE-2 study 30 % of patients received insulin [37]. However, in the Badalona study, in which the annual cost was lower (€2229), the proportion of patients receiving insulin was intermediate (22.3 %) [19]. Of note, many studies omit data on drugs other than insulin and oral antidiabetic agents. However, polypharmacy is a frequent phenomenon among diabetic patients and becomes more frequent with age and the long-term evolution of the disease. Moreover, almost all diabetic patients require not only hypoglycaemic treatment but also treatment for comorbidities and cardiovascular risk factors linked to diabetes. Our study confirms this increase in the cost of other drugs (e.g., cardiovascular drugs) in diabetic patients compared with non-diabetic subjects.

The methodological strengths of this study include the use of a very large sample, the high quality data available from a population database, and the fact that diabetic and non-diabetic patients were individually matched and managed in the same institution by the same physician, meaning that the two groups were fully comparable. Although there might have been estimation errors, these would affect both groups equally, so that the conclusions of the study would not be affected as regards the comparison between groups. Finally, in Spain, patients with T2DM are controlled mainly by primary care professionals, which together with the gatekeeping role played by Spanish general practitioners [38], means that the information available from primary care is very comprehensive. Nevertheless, we want to remark that 14.131 subjects in the non-diabetic group and 2 patients in the diabetic group had nominally no costs. This striking difference may be explained by the fact that since the Spanish health care system is publicly funded, diabetic patients could tend to use more health resources (e.g., material to manage the disease, follow-up visits, quarterly blood analyses, annual reviews to screen for complications) than non-diabetic subjects. Moreover, this is a real-life study and it is conceivable that non-diabetic subjects are healthier (e.g., have less comorbid conditions) than diabetic patients. To avoid producing a bias, especially because this is a controlled study, we used a hurdle model to take into account the difference in the number of zero direct medical cost observations between diabetic and non-diabetic subjects.

One limitation of this study, although common to all studies using population-based databases, is the incompleteness of some clinical variables. For example, data on HbA1c were not available for about 25 % of patients, a shortcoming that may have influenced the actual costs with regard to glycaemic control. In relation to the possible limitation of using the SIDIAP-Q database instead of the whole database, it has been found previously that this subset of patients is representative of the entire population of Catalonia in terms of geographic and demographic characteristics [23, 24]. One of the main limitations of our study is that the costs of medical emergencies, including hypoglycaemia, were not recorded. As the estimated cost of an episode of severe hypoglycaemia may be as high as €3500 [10], the increase in costs found in our study (1.77 times) may be underestimated. In addition, the study design excluded individuals who died during the study period. It is well known that mortality rates in diabetic patients are higher than in the general population [1]. As long as diabetic patients need a comparatively more intensive and expensive use of healthcare resources their inclusion could have produced further incremental costs. Finally, this study reports how incremental costs attributable to T2DM in the context of a public health system are distributed, but we did not assess the impact of indirect costs to society through the quantification of lost productivity by temporary disability or costs due to premature retirement or death, which may also involve a substantial increase in total expenditures. Estimations of indirect costs are not often performed, and findings diverge between studies, ranging between 30 % and 55 % of total costs [3, 5, 6]. Similarly, intangible costs connected to diabetes such as reduced quality of life, need of caregivers or family burden were not included. In chronic diseases, these costs are often significant, but quantifying them is very complex, and they are not included in the majority of studies.

Our results have implications for National Health Service planners, as the increased prevalence of the disease in developed countries is mainly due to population aging, obesity, and the improved survival of people with diabetes [1–4]. Healthcare costs associated with diabetes will increase steadily in the coming years, and will have a profound impact on society and on National Health System expenditures. This trend has been highlighted in several studies and should be taken into account by decision makers when allocating health resources.

In Spain, the number of people with diabetes in 2011 was estimated to be slightly less than 3 million (a prevalence of 7.8 %) [1–3, 39]. As the mean cost of caring for a patient with diabetes in our study was €3110.1, this means that the costs of T2DM are approximately €10 billion (2,917,929 T2DM patients × €3110.1). If we consider that the costs of patients of the same age and sex without diabetes amount to €1803.6, we obtain a figure of €1306 per patient attributable to T2DM; therefore, the additional annual cost of diabetes for those 3 million patients, amounts to €3.9 billions.

## Conclusion

In conclusion, the estimated higher costs for patients with T2DM compared with non-diabetic subjects (72.4 %) are due mainly to hospitalizations and medications, and are higher among diabetic patients with poor glycaemic control and macrovascular complications. The variables with the greatest impact on costs were hospitalization, medication, and visits to primary care centres. Hospitalization and medication generated approximately 70 % of the difference in annual costs between the two groups.

## Electronic supplementary material

Below is the link to the electronic supplementary material.

Supplementary material 1 (DOCX 97 kb)
