# Supplementary material for: Direct medical costs attributable to type 2 diabetes mellitus: a population-based study in Catalonia, Spain
Source: Eur J Health Econ. 2015 Nov 5;17(8):1001–10. doi: 10.1007/s10198-015-0742-5 (PMC5047944; doi:10.1007/s10198-015-0742-5)
Supplement: Supplementary file 1 — Supplementary material 1 (DOCX 97 kb) [file 10198_2015_742_MOESM1_ESM.docx]

*European Journal of Health Economics*

**Cost attributable to type 2 diabetes mellitus: a population-based study in Catalonia, Spain**

Manel Mata-Cases* • Marc Casajuana* • Josep Franch-Nadal • Aina Casellas • Conxa Castell • Irene Vinagre • Dídac Mauricio• Bonaventura Bolíbar

**Corresponding author**:

Dídac Mauricio

Department of Endocrinology & Nutrition, Health Sciences Research Institute

University Hospital Germans Trias i Pujol

Email: didacmauricio@gmail.com

**Figure 1.** Evolution of costs in patients with type 2 diabetes or without diabetes according to age

**Table 1.** Sources of information and costs used in the study

| **Resource** | **Source of information** | **Source of cost** | **Obtained cost** |
| --- | --- | --- | --- |
| **Visits to health professionals**  **(physicians and nurses)** | SIDIAP | DOGC | Physician  Doctor’s office: €40  Home consultation: €65  Nurse  Nurse’s office: €28  Home consultation: €45 |
|  |  | Oblikue database* | Physician  Doctor’s office: €10.50  Home consultation: €27.48  Nurse  Nurse’s office: €10.03  Home consultation: €25.37 |
| **Referrals to specialists** | SIDIAP | DOGC | First visit: €118  Follow-up visit: €51 |
| **Diagnostic tests** | SIDIAP | DOGC | Depending on the requested test |
| **Laboratory analyses** | SIDIAP | DOGC | Depending on the requested item |
| **Consumption of self-monitoring test strips** | SIDIAP | SIDIAP | Total primary care center expenditure divided by the number of type 2 patients |
| **Hospitalizations** | SIDIAP | DRG /DOGC | Depending on the DRG code and hospital’s complexity |
| **Pharmacological treatment** | SIDIAP | Retail price for the consumer | Depending on the Estimated Retail Price (ERP) |
| **Dialysis treatment** | SIDIAP | DOGC | Price of a single session €180 |

*Alternate private costs of primary care visits used in the sensitivity analysis

SIDIAP, Sistema de Información para el Desarrollo de la Investigación en Atención Primaria; DOGC, Diari Oficial de la Generalitat de Catalunya; INE, Instituto Nacional de Estadística

**Table 2.** Mean costs (95% CI) in the study population according to gender

|  | **Patients with type 2 diabetes**  **(n = 126,811)** | | **Patients without diabetes**  **(n = 126,811)** | | **Difference between groups** | |
| --- | --- | --- | --- | --- | --- | --- |
|  | **Men**  **(n = 67,861)** | **Women**  **(n = 58,950)** | **Men**  **(n = 67,861)** | **Women**  **(n = 58,950)** | **Men**  **(n = 67,861)** | **Women**  **(n = 58,950)** |
| **Total annual cost, €** | 3,143.8  (3,080.2-3,207.4) | 3,071.2  (3,010.5-3,132.0) | 1,803.8  (1,755.7-1,851.9) | 1,803.0  (1,726.5-1,879.5) | 1,340.0  (1,260.2-1,419.7) | 1,290.9  (1,170.6-1,365.9) |
| **Primary care visits, €** | 524.5  (520.6-528.5) | 637.3  (632.6-642.1) | 326.7  (323.6-329.8) | 417.8  (414.2-421.5) | 197.8  (192.2 - 202.8) | 219.5  (213.5 - 225.5) |
| **Hospitalizations, €** | 1,444.1  (1,385.2-1,503.1) | 1,140.5  (1,084.1–1,196.9) | 897.1  (851.5–942.8) | 691.6  (616.5–766.8) | 246.9  (472.6 - 621.6) | 448.9  (354.9 - 542.9) |
| **Referrals, €** | 103.8  (102.7-105.0) | 127.5  (126.1–128.9) | 69.1  (68.1–70.0) | 87.3  (86.2–88.4) | 34.8  (33.31 - 36.2) | 40.2  (38.41 - 41.97) |
| **Diagnostic tests, €** | 77.9  (76.9-78.7) | 87.7  (86.6-88.5) | 42.3  (41.6-42.9) | 51.1  (50.2-51.8) | 35.6  (34.5-36.7) | 36.6  (35.3-37.9) |
| **Self-monitoring test strips, €** | 50.1  (49.9-50.1) | 50.0  (50.0-50.2) | N/A | N/A | 50.1  (50.0- 50.2) | 50.0  (49.9- 50.1) |
| **Medication, €** | 873.2  (865.2-881.1) | 984.7  (976.2-993.2) | 448.0  (442.2-453.8) | 536.7  (530.5 - 542.9) | 425.1  (415.3 - 435.0) | 448.0  (437.5 - 458.5) |
| **Dialysis, €** | 70.1  (59.8-80.3) | 43.6  (34.9-52.3) | 20.7  (15.0-26.4) | 18.5  (12.9-24.0) | 49.4  (37.6 - 61.1) | 25.1  (14.8 - 35.5) |

**Table 3.** Mean costs (95% CI) in the study population according to age

|  | **Patients with type 2 diabetes**  **(n = 126,811)** | | | | | **Patients without diabetes**  **(n = 126,811)** | | | | |
| --- | --- | --- | --- | --- | --- | --- | --- | --- | --- | --- |
|  | **≤45 years (n = 5,200)** | **46-55 years**  **(n = 15,494)** | **56-65 years**  **(n = 31,824)** | **66-80 years (n =56,133)** | **≥80 years**  **(n = 18,160)** | **≤45 years (n = 5,467)** | **46-55 years**  **(n = 15,655)** | **56-65 years**  **(n = 31,941)** | **66-80 years (n = 55,915)** | **≥80 years**  **(n = 17,833)** |
| **Total annual cost, €** | 1,497.6  (1,379.3-1,616.0) | 1,971.8  (1,883.4-2,060.2) | 2,630.4  (2,550.7-2,710.2) | 3,607.4  (3,529.7-3,685.1) | 3,846.2  (3,743.3-3,949.2) | 423.7  (387.1 – 460.3) | 769.0  (705.8-832.2) | 1,264.6  (1,206.6-1,322.5) | 2,246.4  (2,159.2-2,333.6) | 2,710.9  (2,621.8-2,800.1) |
| **Primary care visits, €** | 374.1  (363.5-384.6) | 409.6  (403.0-416.1) | 480.5  (475.7-485.2) | 634.5  (629.8-639.3) | 769.1  (758.4-779.8) | 142.3  (136.3-148.3) | 190.2  (186.2-194.2) | 273.0  (269.7-276.3) | 427.2  (423.6-430.8) | 585.4  (576.4-594.3) |
| **Hospitalizations, €** | 477.7  (368.6-586.7) | 715.8  (634.8-796.9) | 1,048.6  (974.5-1,122.7) | 1,554.6 (1,482.1-1,627.0) | 1,708.7 (1,613.4-1,804.0) | 142.1  (111.6-172.6) | 339.8  (278.8-400.9) | 548.1  (492.5-603.5) | 1,021.2  (935.4-1,106.9) | 1,174.7  (1,093.2-1,256.2) |
| **Referrals, €** | 83.7  (79.9-87.4) | 95.1  (92.8-97.4) | 109.9  (108.2-111.6) | 127.5  (126.1-128.9) | 110.1  (107.7-110.2) | 39.2  (36.8-41.5) | 51.5  (49.9-53.2) | 68.9  (67.6-70.2) | 91.4  (90.3-92.6) | 84.1  (82.1-86.1) |
| **Diagnostic tests, €** | 69.4  (66.8-71.9) | 73.7  (72.3-75.2) | 81.4  (80.4-82.4) | 88.1  (87.3-88.9) | 77.5  (76.2-78.8) | 22.4  (20.8-24.0) | 29.6  (28.6-30.7) | 42.7  (41.9-43.5) | 53.4  (52.7-54.1) | 52.7  (51.5-53.9) |
| **Self-monitoring test strips, €** | 48.8  (48.5-49.2) | 49.6  (49.5-49.8) | 50.2  (50.1-50.4) | 50.2  (50.1-50.3) | 49.9  (49.8-50.1) | -- | -- | -- | -- | -- |
| **Medication, €** | 430.7  (407.8-453.5) | 599.3  (583.7- 615.0) | 822.7  (811.0- 834.3) | 1,073.1  (1,064.2- 1,081.9) | 1,066.0 (1,051.8-1,080.2) | 76.6  (66.0- 87.3) | 154.1  (146.8- 161.4) | 323.1  (316.0- 330.2) | 626.5  (619.6- 633.4) | 777.1  (764.2- 789.8) |
| **Dialysis, €** | 13.4  (-2.4-29.2) | 28.6  (15.2-42.0) | 37.0  (26.2-47.9) | 79.4  (65.4-91.4) | 64.8  (45.7-83.9) | 1.09  (-1.0 - 3.2) | 3.59  (-1.4 – 8.7) | 8.77  (3.7-13.9) | 26.65  (19.6-33.7) | 37.0  (22.2-51.8) |

**Table 4.** Mean costs (95% CI) in the study population according to the main pathologies related with diabetes

|  | **Patients with type 2 diabetes**  **(n = 126,811)** | | **Patients without diabetes**  **(n = 126,811)** | | **Difference between groups** | |
| --- | --- | --- | --- | --- | --- | --- |
|  | **With pathology** | **Without pathology** | **With pathology** | **Without pathology** | **With pathology** | **Without pathology** |
| **Coronary artery disease**, €  With type 2 diabetes: n = 16,077  Without type 2 diabetes: n = 7,878 | 5,006.3  (4,842.5-5,170.2) | 2,834.8  (2,790.3-2,879.2) | 3,381.8  (3,200.5-3,563.1) | 1,698.9  (1,653.7-1,744.1) | 1,624.2  (1,358.3-1,890.8) | 1,135.9  (1,072.3-1,199.4) |
| **Stroke**, €  With type 2 diabetes: n = 9,580  Without type 2 diabetes: n = 5,777 | 4,720.1  (4,535.8-4,904.4) | 2,978.5  (2,933.2-3,023.8) | 3,145.5  (2,969.8-3,321.3) | 1,739.4  (1,694.2 – 1,784.6) | 1,574.5  (1,300.7 – 1,848.3) | 1,239.1  (1,175.1 – 1,303.1) |
| **Peripheral artery disease**, €  With type 2 diabetes: n = 5,848  Without type 2 diabetes: n = 2,246 | 5,858.3  (5,462.0-6,254.6) | 2,977.3  (2,935.2-3,019.3) | 4,178.9  (3,739.3-4,618.4) | 1,760.6  (1,716.7-1,804.6) | 1,679.4  (1,087.7-2,271.2) | 1,216.7  (1,155.8-1,277.5) |
| **Heart failure**, €  With type 2 diabetes: n = 7,298  Without type 2 diabetes: n = 521 | 6,866.6  (6,563.1-7,170.1) | 2,880.7  (2,837.9-2,923.4) | 4,759.2  (4,422.6-5,095.8) | 1,719.0  (1,675.0-1,763.1) | 2,107.4  (1,654.2-2,560.6) | 1,161.6  (1,100.3-1,223.0) |
| **Diabetic retinopathy**, €  (n = 9,132) | 4,700.7  (4,503.3-4,898.0) | 2,986.6  (2,941.6-3,031.7) | N/A | N/A | N/A | N/A |
| **Diabetic neuropathy**, €  (n = 12,512) | 4,273.3  (4,096.7-4,449.8) | 2,699.3  (2,653.1-2,745.5) | N/A | N/A | N/A | N/A |
| **Chronic renal failure**, €  With type 2 diabetes: n = 15,780  Without type 2 diabetes: n = 9,958 | 4,691.6  (4,532.6-4,850.7) | 2,727.3  (2,687.3-2,767.3) | 3,163.5  (2,788.0-3,539.0) | 1,910.7  (1,868.3-1,953.1) | 1528.1  (1,120.3-1,935.9) | 816.6  (758.3-875.0) |
| **Microalbuminuria**^a^**,** €  With type 2 diabetes: n = 7,990  Without type 2 diabetes: n = 1,361 | 3,938.9  (3,772.8-4,105.0) | 2,699.3  (2,653.1-2,745.5) | 4,799.7  (1,939.8-7,659.7) | 1,994.3  (1,933.0-2,055.6) | -860.8  (-3,725.6-2,004.0)* | 705.0  (628.3- 781.7) |
| **Macroalbuminuria**^b^, €  With type 2 diabetes: n = 1,236  Without type 2 diabetes: n = 131 | 6,434.5  (5,680.1-7,189.0) | 2,699.3  (2,653.1-2,745.5) | 6,995.3  (4,464.9-9,525.6) | 1,994.3  (1,933.0-2,055.6) | -560.7  (-3,199.0 – 2,077.6)* | 705.0  (628.3- 781.7) |
| **Any microvascular disease**^c^, €  With type 2 diabetes: n = 5,779  Without type 2 diabetes: n = 11 | 5,245.8  (4,964.2-5,527.4) | 3,008.1  (2,963.8-3,052.3) | 7,713.0  (-2,163.6-17,589.6) | 1,802.9  (1,759.1-1,846.8) | -2,467,2  (-12,346.2-7,411.8)* | 1,205.2  (1,142.8-1,267.5) |
| **Any cardiovascular disease**^d^, €  With type 2 diabetes: n = 27,331  Without type 2 diabetes: n = 14,301 | 4,815.6  (4,683.7-4,947.4) | 2,648.8  (3,605.8 1-2,691.8) | 3,306.8  (3,173.0-3,440.6) | 1,612.4  (1,566.0-1,658.7) | 1,508.8  (1,320.9-1,696.7) | 1,036.4  (973.2-1,099.6) |

*No statistically significant differences

^a^Albuminuria between 30 mg/mL and 300 mg/mL

^b^Albuminuria >300 mg/mL

^c^Patients with diabetic retinopathy, diabetic nephropathy (micro or macroalbuminuria), and diabetic neuropathy

^d^Patients with peripheral artery disease, stroke, and coronary heart disease

**Table 5.** Multivariate analysis comparing patients with type 2 diabetes or without diabetes using a hurdle model

| **Count model** | **Unadjusted IRR** | | **IRR adjusted for age and gender** | | **IRR adjusted for age, gender, cardiovascular disease, and heart failure** | | **IRR adjusted for age, gender, cardiovascular disease, heart failure, and end-stage kidney disease** | |
| --- | --- | --- | --- | --- | --- | --- | --- | --- |
|  | IRR (95% CI) | p-value | IRR (95% CI) | p-value | IRR (95% CI) | p-value | IRR (95% CI) | p-value |
| **Type 2 diabetes** |  |  |  |  |  |  |  |  |
| No |  |  | -- |  | -- |  | -- |  |
| Yes | 1.54 (1.53-1.56) | <0.001 | 1.63 (1.61-1.65) | <0.001 | 1.51 (1.50-1.22) | <0.001 | 1.41 (1.38-1.44) | <0.001 |
| **Age, years** |  |  |  |  |  |  |  |  |
| <46 |  |  | -- |  | -- |  | -- |  |
| 46-55 |  |  | 1.42 (1.38-1.46) | <0.001 | 1.37 (1.33-1.41) | <0.001 | 1.36 (1.24-49) | <0.001 |
| 56-65 |  |  | 1.94 (1.89-1.99) | <0.001 | 1.81 (1.76-1.85) | <0.001 | 1.59 (1.46-1.74) | <0.001 |
| 66-80 |  |  | 2.93 (2.86-3.01) | <0.001 | 2.54 (2.48-2.61) | <0.001 | 2.03 (1.87-2.21) | <0.001 |
| >80 |  |  | 3.36 (3.27-3.45) | <0.001 | 2.70 (2.63-2.77) | <0.001 | 1.95 (1.79-2.13) | <0.001 |
| **Gender** |  |  |  |  |  |  |  |  |
| Men |  |  | -- |  | -- |  | -- |  |
| Women |  |  | 0.90 (0.89-0.91) | <0.001 | 0.95 (0.94-0.96) | <0.001 | 0.95 (0.93-0.97) | <0.001 |
| Cardiovascular disease^a^ |  |  |  |  | 1.54 (1.52-1.56) | <0.001 | 1.71 (1.66-1.78) | <0.001 |
| Heart failure |  |  |  |  | 1.92 (1.88-1.97) | <0.001 | 1.37 (1.34-1.40) | <0.001 |
| End-stage kidney disease^b^ |  |  |  |  |  |  | 1.79 (1.75-1.82) | <0.001 |

IRR, incident rate ratio

^a^Includes peripheral artery disease, stroke, and coronary heart disease

^b^Includes dialysis, transplantation, and/or estimated Glomerular Filtration Rate (MDRD formula) <15 mL/min/1.73 m^2^

**Table 6**. Increase of healthcare costs in type 2 diabetic vs. non-diabetic patients. Figures are updated to 2011 € values

|  | **T2DM** | **Non T2DM** | **Difference** | **Ratio** |
| --- | --- | --- | --- | --- |
| Koster et al. (Germany, 2001)^16^ | 6,932 | 3,629 | 3,303 | 1.9 |
| Bruno et al. (Italy, 2003)^14^ | 4,519 | 1,106 | 3,413 | 4.1 |
| Wiréhn et al. (Sweden, 2005)^13^ | 5,161 | 2,888 | 2,273 | 1.8 |
| ADA (US, 2007)^18^ | 7,127 | 1,766 | 5,361 | 3 |
| ADA (US, 2012)^5^ | 8,117 | 2,065 | 6,052 | 2.9 |
| Koster et al. (Germany, 2010)^17^ | 5,191 | 2750 | 2,441 | 1.9 |
| SIDIAP (Spain, 2011) | 3,101 | 1,804 | 1,297 | 1.7 |
